# Assessment of Spatiotemporal Fusion Algorithms for Planet and Worldview Images

**DOI:** 10.3390/s18041051

**Published:** 2018-03-31

**Authors:** Chiman Kwan, Xiaolin Zhu, Feng Gao, Bryan Chou, Daniel Perez, Jiang Li, Yuzhong Shen, Krzysztof Koperski, Giovanni Marchisio

**Affiliations:** 1 Applied Research LLC, Rockville, MD 20850, USA; choub90@gmail.com; 2 Department of Land Surveying and Geo-Informatics, The Hong Kong Polytechnic University, Kowloon, Hong Kong 999077, China; xiaolin.zhu@polyu.edu.hk; 3 Hydrology & Remote Sensing Lab, USDA ARS, Beltsville, MD 20704, USA; feng.gao@ars.usda.gov; 4 Department of Electrical & Computer Engineering, Old Dominion University, Norfolk, VA 23529, USA; dpere013@odu.edu (D.P.); jli@odu.edu (J.L.); yshen@odu.edu (Y.S.); 5 Digital Globe, Inc., Herndon, VA 20171, USA; kkopersk@digitalglobe.com (K.K.); giovanni.marchisio@comcast.net (G.M.)

**Keywords:** image fusion, Planet, Worldview, pansharpening, forward prediction, spatiotemporal

## Abstract

Although Worldview-2 (WV) images (non-pansharpened) have 2-m resolution, the re-visit times for the same areas may be seven days or more. In contrast, Planet images are collected using small satellites that can cover the whole Earth almost daily. However, the resolution of Planet images is 3.125 m. It would be ideal to fuse these two satellites images to generate high spatial resolution (2 m) and high temporal resolution (1 or 2 days) images for applications such as damage assessment, border monitoring, etc. that require quick decisions. In this paper, we evaluate three approaches to fusing Worldview (WV) and Planet images. These approaches are known as Spatial and Temporal Adaptive Reflectance Fusion Model (STARFM), Flexible Spatiotemporal Data Fusion (FSDAF), and Hybrid Color Mapping (HCM), which have been applied to the fusion of MODIS and Landsat images in recent years. Experimental results using actual Planet and Worldview images demonstrated that the three aforementioned approaches have comparable performance and can all generate high quality prediction images.

## 1. Introduction

Due to high spatial resolution, Worldview-2 (WV) images have been widely used for hazard assessment, nuclear site monitoring, border activity detection, etc. One issue is the revisit time of WV satellite. On the other hand, although the resolution of Planet images is lower than that of WV, Planet images use multiple small satellites to collect images and can cover the same area almost daily. It would be ideal to fuse the above images to create a high spatial and high temporal resolution image sequence so that quick and responsive actions can be taken for certain applications such as damage assessment due to hurricanes, fires, tsunamis, etc.

One may question the necessity of this research, as the spatial resolutions of WV (2 m) and Planet (3.125 m) images are all of high resolution and do not seem to differ that much. A simple bicubic interpolation of Planet images should be sufficient. It turns out that, as can be seen in Section 2 and Section 3, the visual appearance of Planet images seems to be much worse than 3.125 m. We interacted with Planet engineers and inquired about why the Planet images do not seem to have 3.125 m resolution, but we could not get any direct feedback. We speculated that there might be some smearing effects due to various point spread functions [1,2] in the imaging hardware in Planet satellites.

It is also important to emphasize that the goal of our research is very different from traditional remote sensing applications such as vegetation monitoring, forest monitoring, etc. Our goal is to perform border monitoring, which includes monitoring of buildings, military built-up, and road construction near borders, illegal trail detection, illegal tunnel digging activity detection, etc., using high resolution satellite images. Conventional remote sensing platforms such as Landsat (30 m resolution, 16-day revisit time) and MODIS (500 m resolution and almost daily revisit) do not have enough spatial resolution to meet our sponsor’s needs. Therefore, we have looked at Worldview and Planet images. After some investigations, we found that the Planet images’ resolution is not as good as it should be. Consequently, we decided to embark on this research effort and see if we could generate high temporal and high spatial resolution images by fusing Worldview and Planet images.

There have been on-going research activities in fusing MODIS and Landsat images throughout the past decade. In [3], a fusion approach known as Spatial and Temporal Adaptive Reflectance Fusion Model (STARFM) was proposed and demonstrated. Several alternative algorithms [4,5,6] were published to further improve the fusion performance. According to a review paper [7], the Spatial Temporal Adaptive Algorithm for mapping Reflectance Change (STAARCH) [4] approach can handle abrupt changes, but requires two pairs of MODIS and Landsat images. The enhanced spatial and temporal adaptive reflectance fusion model (ESTARFM) [5] algorithm focuses on enhancing performance of mixed pixels. Similar to STAARCH, ESTARFM requires two pairs of images. As a result, both STAARCH and ESTARFM may not be suitable for forward prediction, i.e., using the images in the past to predict the image at the future time. Recently, two approaches have been proposed for fusing MODIS and Landsat. In [8], Flexible Spatiotemporal Data Fusion (FSDAF) was proposed, which can handle heterogeneous images very well. In [9], the Hybrid Color Mapping (HCM) approach was proposed for fusing MODIS and Landsat images and the idea works well for homogeneous images. In addition to the above papers, there are other fusion approaches in the literatures. See [10,11,12,13,14,15] and references therein.

Pansharpening refers to the use of a high resolution panchromatic (pan) band to sharpen low resolution multispectral bands. Many approaches have been proposed in the past two decades. In recent years, new pansharpening approaches have been developed that can utilize high resolution multispectral bands for pansharpening. See [16,17] and references therein. After a careful study of the fusion problem between WV and Planet, it turns out that pansharpening algorithms, such as the algorithms mentioned in [16,17], cannot be applied. The main reason is that most existing pansharpening algorithms require low resolution multi-spectral images and a high resolution pan image at the time of prediction. In the fusion of WV and Plant images, this is not possible. We have actually applied several pansharpening algorithms such as Gram-Schmidt Adaptive (GSA), Principal Component Analysis (PCA), etc. in [16] to enhance a MODIS image at time *t*_2_ by using a better resolution Landsat image at an earlier time *t*_1_. The results are not satisfactory due to severe changes in image contents between the two images.

In this paper, we summarize the results of applying STARFM, FSDAF, and HCM approaches to fusing WV and Planet images for forward prediction. To the best of our knowledge, no one has carried out such a study before for WV and Planet images. The first two approaches, STARFM and FSDAF, are well-known in fusing the MODIS and Landsat community. The third approach (HCM) was motivated by our recent pansharpening work for synthesizing a high resolution hyperspectral image by fusing a high resolution color image with a low resolution hyperspectral image cube [18]. Similar to STARFM and FSDAF, only one pair of Planet and WV images is needed for prediction. Experiments using actual Planet and WV images were used to quantify the performance of the three approaches.

Our paper is organized as follows. Section 2 briefly reviews the three fusion methods. Section 3 presents our experimental results. Finally, conclusions and future research directions will be presented in Section 4.

## 2. Spatiotemporal Fusion Approaches

In the next few sections, we will briefly summarize the STARFM, FSDAF, and HCM algorithms. Both Planet and WV images are co-registered and resampled to the same image size and extent according to the requirement of these fusion algorithms. 

### 2.1. STARFM 

If ground cover type and system errors at pixel (*x*, *y*) have not changed between the observation date $t_{k}$ and the prediction date $t_{p}$, where a WV scene is not available, then the temporal changes of surface reflectance from WV should be equivalent to the temporal changes in the corresponding Planet pixel between two dates. If the bias between WV and Planet stays the same for two dates ($\varepsilon_{p}=\varepsilon_{k}$), we have

(1) $$W(x,y,t_{p})=W(x,y,t_{k})+(P(x,y,t_{p})-P(x,y,t_{k}))$$

where *W*, *P* denote pixels in WV and Planet images, respectively.

To address mixed pixels in the prediction, STARFM [3,7] introduces additional information from neighboring pixels and uses spectrally similar pixels for prediction. That is, the predicted surface reflectance for the central pixel at date $t_{p}$ is computed with a weighting function from spectrally similar neighboring pixels within a specified search window:

(2) $$W(w/2,w/2,t_{p})=\sum_{k=1}^{M}\sum_{i=1}^{N}\alpha_{ik}\times \left(W(x,y,t_{k})+(P(x,y,t_{p})-P(x,y,t_{k}))\right)$$

where *w* is the searching window size and (*w*/2, *w*/2) is the central pixel of this moving window. The size of the searching window was determined as three coarse resolution pixels. In this paper, the size of the searching window is defined as 12 m (±6 m searching distance), which covers about three Planet pixels. *N* represents the total number of similar Planet pixels in the moving window and *i* is the index. *M* represents the total number of pairs of observed Planet and WV images. The weighting function $\alpha_{ik}$ determines how much each neighboring pixel, *i*, contributes to the estimated reflectance of the central pixel from image pair *k*. Mathematically the weighting α_*ik*_ is given by

(3) $$\alpha_{ik}=(1.0/C_{ik})/\sum_{i=1}^{N}\left(1.0/C_{ik}\right)\;C_{ik}=\left|P(x,y,t_{k})-P(x,y,t_{p})\right|*\left|P(x,y,t_{k})-W(x,y,t_{k})\right|*D_{i}$$

where *D_i_* is the distance between candidate pixel (*i*) at location (*x*,*y*) and the central pixel of the moving window at location (*w*/2, *w*/2). It is determined by three measures based on: (1) spectral difference between the Planet and WV data at a given location; (2) temporal difference between the input Planet data ($P(x,y,t_{k})$) and the Planet data at the prediction date ($P(x,y,t_{p})$); and (3) the geographic distance between the central pixel and the candidate pixel. Closer pixels are weighted more. Pixels with smaller spectral and temporal differences also receive higher weight. Weights for all spectrally similar pixels are normalized (so the sum of weights equals one) before applying to Equation (2). These measures ensure that pure and close neighbor pixels get higher weights in the prediction.

### 2.2. FSDAF 

**Figure 1:**
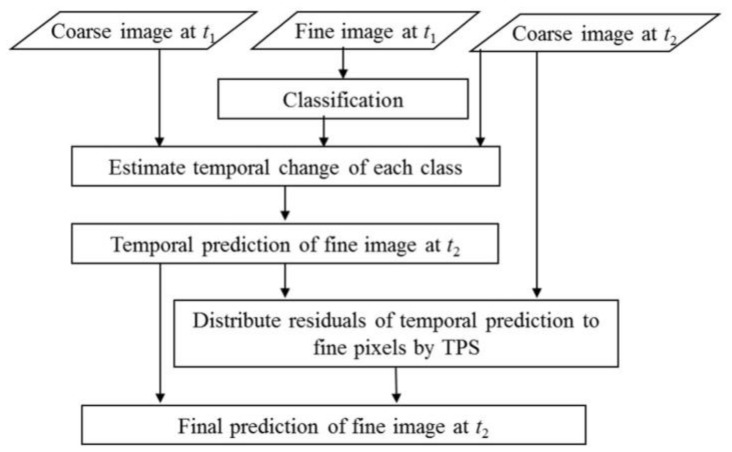
Flowchart of the Flexible Spatiotemporal Data Fusion (FSDAF) fusion algorithm. Figure reproduced from [8].

In FSDAF [8], the input data include one pair of coarse and fine resolution images acquired at *t*_1_ and one coarse-resolution image at *t*_2_ (Figure 1). The output is a predicted fine-resolution image at *t*_2_. In this study, the coarse-resolution image is Planet image while the fine-resolution image is WV image. FSDAF includes six main steps: (1) classify the fine-resolution image at *t*_1_ by an unsupervised classifier, Iterative Self-Organizing Data Analysis Technique Algorithm (ISODATA) [19]; (2) estimate the temporal change of each class in the coarse-resolution image from *t*_1_ to *t*_2_; (3) predict the fine-resolution image at *t*_2_ using the class-level temporal change (refereed as temporal prediction) and calculate residuals at each coarse pixel. This temporal prediction would be accurate if no abrupt land cover change occurs; (4) predict the fine-resolution image from the coarse image at *t*_2_ with a Thin Plate Spline (TPS) interpolator. This TPS prediction should be more accurate than the prediction in the previous step if land cover change occurs; (5) distribute the residuals in step 3 based on TPS prediction to update the temporal prediction; and (6) further smooth the temporal prediction to get the final prediction of the fine-resolution image at *t*_2_ using the similar strategy of STARFM. The number of classes used in Step 2 is based on the output of ISODATA. In ISODATA, users need to set the minimum and maximum number of classes based on their prior knowledge of the image, and then ISODATA will find the optimal number of classes. In Step 6, FSDAF smooths the temporal prediction using the weighted average of the same-class pixels in a neighborhood. The weight is determined by spatial distance. More details can be found in the paper introducing FSDAF [8].

### 2.3. Hybrid Color Mapping (HCM) Approach 

**Figure 2:**
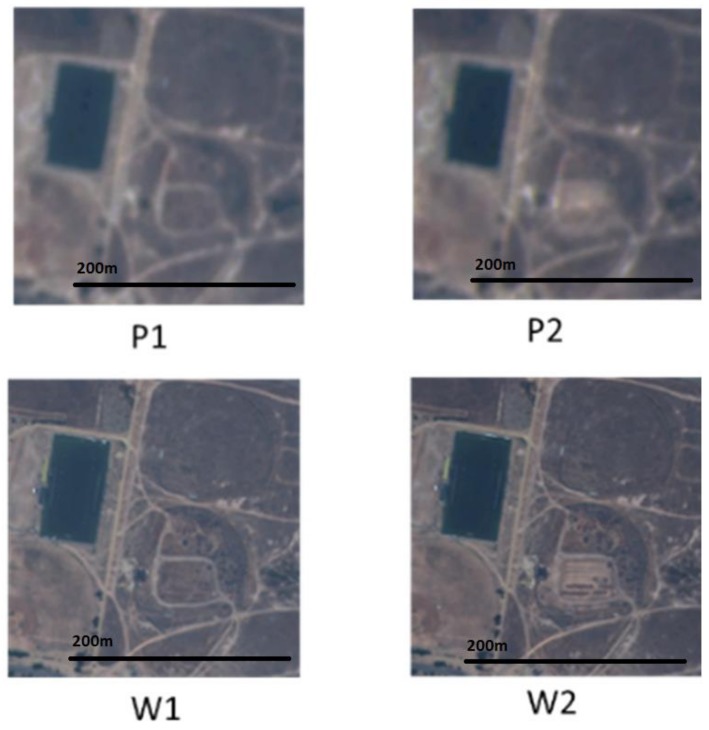
Relationship between two pairs of Planet (top row) and Worldview-2 (WV) (bottom row) images.

We will use WV and Planet images to illustrate the HCM approach [9]. Figure 2 shows two pairs of Planet (P1 and P2) and WV (W1 and W2) images. The pairs, (P1, W1) and (P2, W2) are collected on the same days. We have two observations. First, the Planet images can be treated as blurred versions of their WV counterparts. Second, the intensity relationship between the Planet pixels is somewhat similar to that of those WV pixels. If we can capture the intensity mapping between Planet images at two different times, then we can use that mapping to predict the WV image at time $t_{p}$ using the WV image at $t_{k}$. It turns out that, although the above idea is simple and intuitive, the prediction results using this idea are quite accurate.

To further justify the above observation, we provide some statistics (means and standard deviations (std)) shown in Table 1, Table 2 and Table 3 for the Planet and Worldview image pairs. PSE and WVE are Planet and Worldview image pair at an earlier time and PSL and WVL are the image pair at a later time. The numbers are quite close.

**Figure 3:**
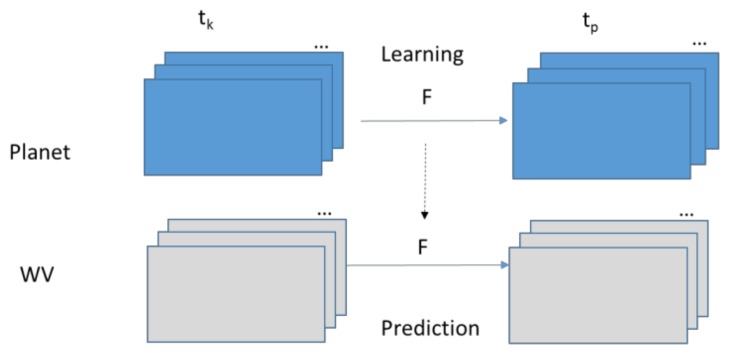
The Hybrid Color Mapping (HCM) approach for image fusion.

Figure 3 illustrates the HCM approach. Based on the available Planet images collected at $t_{k}$ and $t_{p}$, we learn the pixel by pixel mapping between the two images. The learned matrix, *F*, is then applied in the prediction step. Using similar notations in earlier equations, the prediction of the WV image at $t_{p}$ can be achieved by

(4) $$W(x,y,t_{p})=F\times W(x,y,t_{k})$$

where W(·,·,·) denotes a pixel vector (up to *K* with *K* being the number of bands) for this application and F is a pixel to pixel mapping/transformation matrix with appropriate dimensions. F can be determined by using the following relationship:

(5) $$P(x,y,t_{p})=F\times P(x,y,t_{k})$$

where P(·,·,·) denotes a pixel vector (*K* bands). To account for intensity differences between two images, a variant of Equation (4) can be described as

(6) $$P(x,y,t_{p})=F_{1}\times P(x,y,t_{k})+F_{2}$$

where $F_{2}$ is a vector of constants. Procedures to obtain *F* can be found in [9].

**Figure 4:**
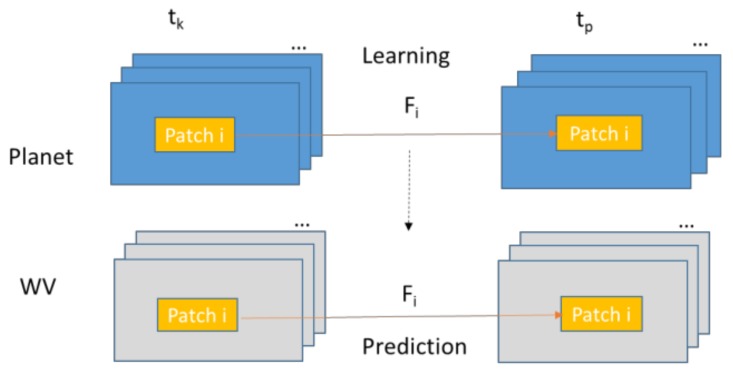
Proposed prediction approach based on local mapping.

Based on our observations, in some cases, prediction results will be more accurate if we divide the images into patches. Each patch will have its own mapping matrix. Figure 4 illustrates the local prediction approach. The patches can be overlapped or non-overlapped. Moreover, for each local patch, which can be a single band or a multi-band image, we use the same estimation algorithm [9] to determine the local mapping matrix, *F_i_*.

### 2.4. Performance Metrics

Although there are many performance metrics in the literature, we selected the following ones: peak signal-to-noise ratio (PSNR) [1], structural similarity (SSIM) [8], Q2N [9], absolution difference (AD) [9], root mean squared error (RMSE) [18], spectral angle mapper (SAM) [18], cross correlation (CC) [18], Erreur Relative Globale Adimensionnelle de Synthese (ERGAS) [18], In addition to the above metrics, we also use SSIM maps to visualize the similarity between two images.

- Absolute Difference (AD). The AD of two vectorized images S (ground truth) and $\hat{S}$ (prediction) is defined as
  (7) $$\mathrm{AD}(S,\hat{S})=\frac{1}{Z}\sum_{j=1}^{Z}\left|s_{j}-{\hat{s}}_{j}\right|$$
  where *Z* is the number of pixels in each image. The ideal value of AD is 0 if the prediction is perfect.
- RMSE (Root Mean Squared Error). The RMSE of two vectorized images S (ground truth) and $\hat{S}$ (prediction) is defined as
  (8) $$\mathrm{RMSE}(S,\hat{S})=\sqrt{\frac{1}{Z}\sum_{j=1}^{Z}{\left(s_{j}-{\hat{s}}_{j}\right)}^{2}}$$
  where *Z* is the number of pixels in each image. The ideal value of RMSE is 0 if the prediction is perfect.
- PSNR (Peak Signal to Noise Ratio). PSNR is related to RMSE defined in (8). If the image pixels are expressed in doubles with values between 0 and 1, then
  (9) $$\mathrm{PSNR}=20\log(1/\mathrm{RMSE}(S,\hat{S}))$$
- CC (Cross-Correlation). We used the codes from Open Remote Sensing website (https://openremotesensing.net/). The ideal value of CC is 1 if the prediction is perfect.
- ERGAS (Erreur Relative. Globale Adimensionnelle de Synthese). The ERGAS is defined as
  (10) $$\mathrm{EGARS}(S,\hat{S})=100d\frac{\mathrm{RMSE}}{\mu }$$
  for some constant *d* depending on the resolution and μ is the mean of the ground truth images. The ideal value of ERGAS is 0 if a prediction algorithm is perfect.
- SSIM (Structural Similarity). This is a metric to reflect the similarity between two images. An equation for SSIM can be found in [8]. The ideal value of SSIM is 1 for perfect prediction. We also use the SSIM map to display the similarity values at each pixel location. Bright pixels have high similarity.
- SAM (Spectral Angle Mapper). The spectral angle mapper measures the angle between two vectors. The ideal value of SAM is 0 for perfect reconstruction. For single bands, the angle is zero between two scalar pixels. The exact definition of SAM can be found in [17].
- Q2N: A definition for Q2N can be found in [17]. The ideal value of Q2N is 1. The codes can be downloaded from Open Remote Sensing website (http://openremotesensing.net/).

## 3. Results

In this section, we present some experimental results. Since our main interest is in forward prediction, we only compare with STARFM and FSDAF, which require one pair of Worldview and Planet images at an earlier time, and one Planet image at the time of prediction. We did not compare with the ESTARFM and STAARCH methods, which, in addition to requiring one Planet image at the prediction time, also require two pairs of Worldview and Planet images: one pair at an earlier time and another pair at a future time. In other words, ESTARFM and STAARCH are suitable for processing archived data, not for forward prediction. 

In order to make our paper self-contained, we include the following specifications of Planet and Worldview images in Table 4. Only four bands have similar spectral ranges.

We used WV-2 images that have eight bands, of which we only used the RGB bands for ease of demonstration/visualization. In addition, because Planet images only have four bands, using the RGB bands in both WV-2 and Planet images will be sufficient for both visualization and performance evaluation. The spatial resolution of WV-2 images is 2 m. Most of the Worldview images are off-nadir images in order to have a large coverage area. Moreover, because there is only one Worldview satellite, the revisit times are not as frequent as the Planet satellite constellation of 120 Cubesats.

Each PlanetScope satellite is a CubeSat 3U form factor (10 cm by 10 cm by 30 cm). The complete PlanetScope constellation of approximately 120 satellites will be able to scan the entire land surface of the Earth every day [20]. In Planet image archive, there are Rapideye and Planetscope images. In this study, we used Planetscope images (orthorectified). The image resolution is 3.125 m. For Planet images, only top of atmosphere radiance (TOAR) data products are available. For WV images, surface reflectance images are available. In order to use compatible data, our collaborators at Digital Globe generated WV TOAR images for this study.

Our area of interest is a border area near Tijuana and San Diego. From data archives of both Planet and Digital Globe, the following images were used in our study:

Planet images: 19 July 2017, 27 July 2017, and 5 August 2017;
WV images: 18 July 2017, 26 July 2017, and 3 August 2017

It should be noted that it is difficult to retrieve Planet and WV images for the same dates. However, this also justifies our research, as our goal is to generate high spatial resolution images when high resolution WV images are not available. It is also emphasized that the registration of the two different types of satellite images is non-trivial, as WV images are not taken at nadir. As a result, automated registration algorithms using corner points from buildings may lead to large registration errors at ground level pixels. In this research, we manually selected ground feature points such as road intersections for image alignment. 

From the above collected images, we focused on three specific sub-areas representing different scenarios.

### 3.1. Scenario 1: Canal Area

**Figure 5:**
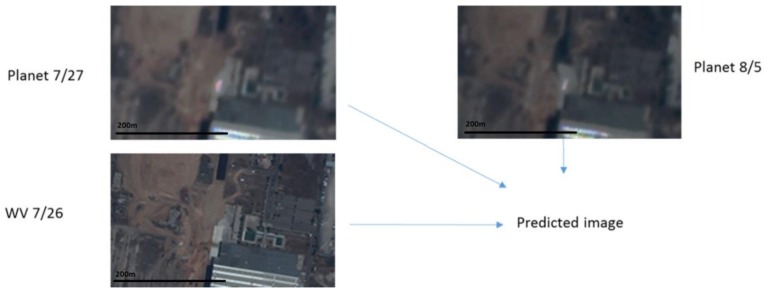
Two Planets images at 27 July and 5 August and one WV image at 26 July are fused to generate a prediction. The prediction is then compared to a WV image collected on 3 August 2017.

**Figure 6:**
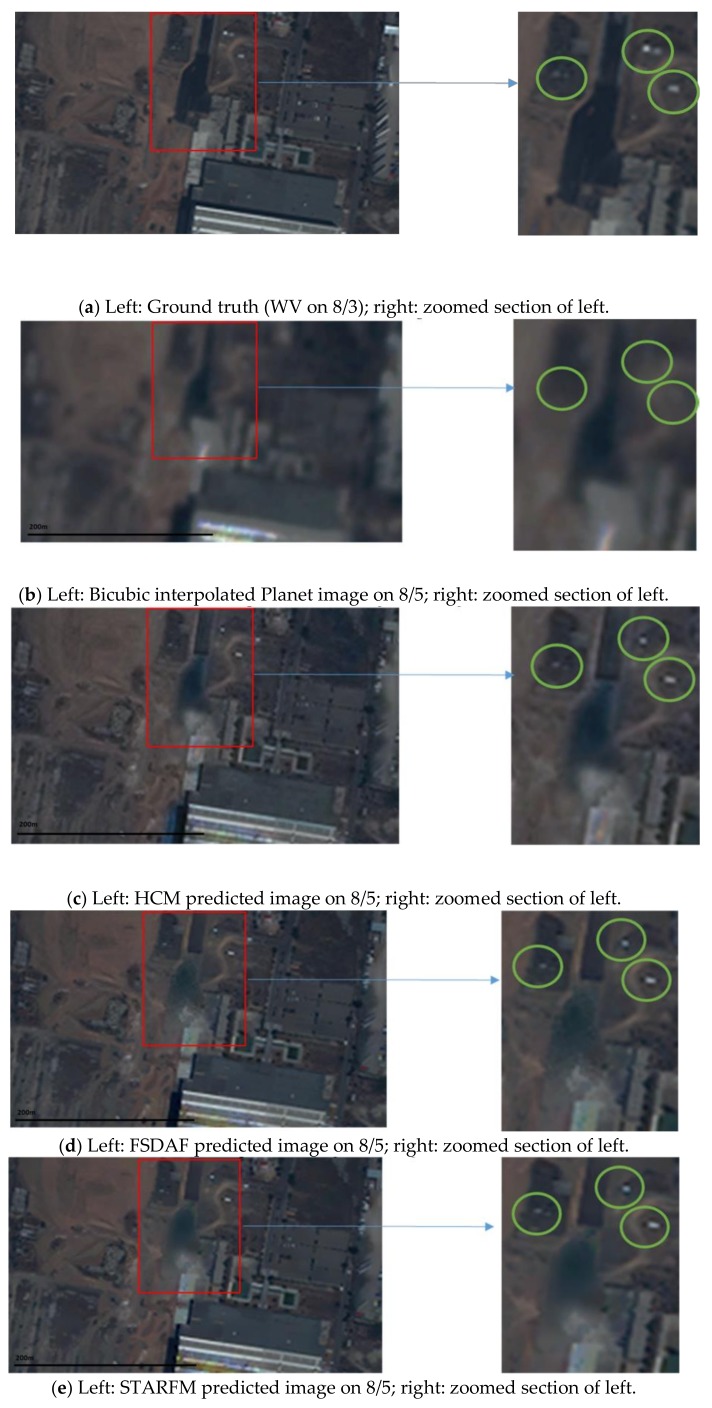
Comparison of different fused images with the ground truth (WV) and the low resolution Planet image at the prediction time. Canal area.

**Figure 7:**
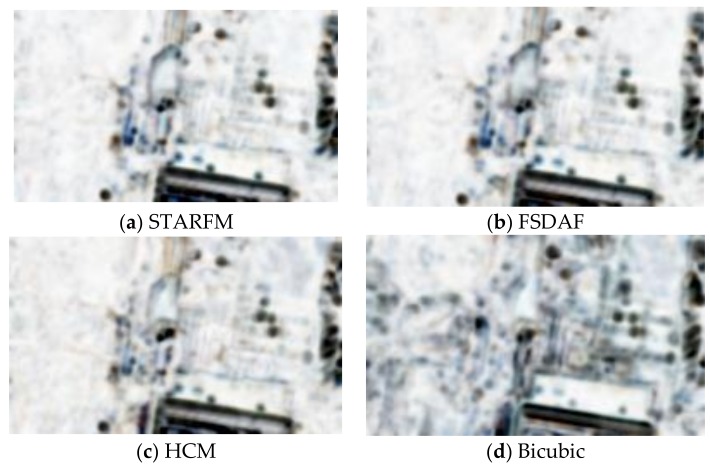
SSIM (Structural Similarity) maps of the Canal scenario using different methods. (**a**–**d**) are the maps for STARFM, FSDAF, HCM, and bicubic, respectively.

In this case, we used two Planet images collected on 27 July 2017 and 5 August 2017 and two WV images collected on 26 July 2017 and 8 August 2017. The prediction scenario is summarized in Figure 5. Eight performance metrics were used. Due to resolution differences between Planet and WV images, we applied bicubic interpolation to upsample the Planet images to the same size of the WV images. Table 5 summarizes the performance metrics for RGB bands as well as individual bands. First, it can be seen that all of the three fusion methods have significant improvement (all metrics) over the Planet image at the prediction time. The PSNR values have been improved by 0.6 dB and the SSIM values improved by more than 0.11. Other metrics all show improvements over those of bicubic. Second, among the three fusion methods, HCM and FSDAF performed slightly better than STARFM. Figure 6 shows visual comparison of the three fused images with the ground truth (WV) and low resolution Planet images at the prediction time. It can be seen that the three algorithms can generate very high quality predictions. In particular, some small objects inside the green circles were correctly predicted using the three algorithms. In Figure 7, we also generated SSIM maps for this scenario. Bright indices show high similarity values. One can see that the HCM, FSDAF, and STARFM all have better SSIM values than that of bicubic interpolation.

### 3.2. Scenario 2: Field Area

**Figure 8:**
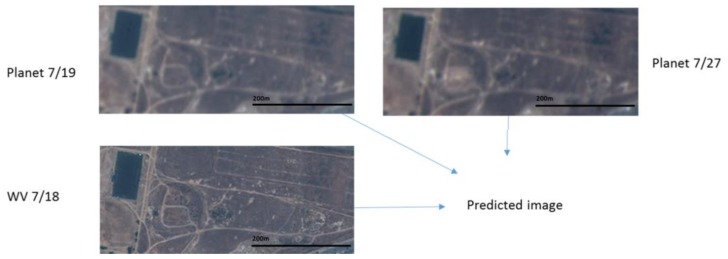
Two Planets images at 19 July and 27 July and one WV image at 18 July are fused to generate a prediction. The prediction is then compared to a WV image collected on 26 July 2017.

**Figure 9:**
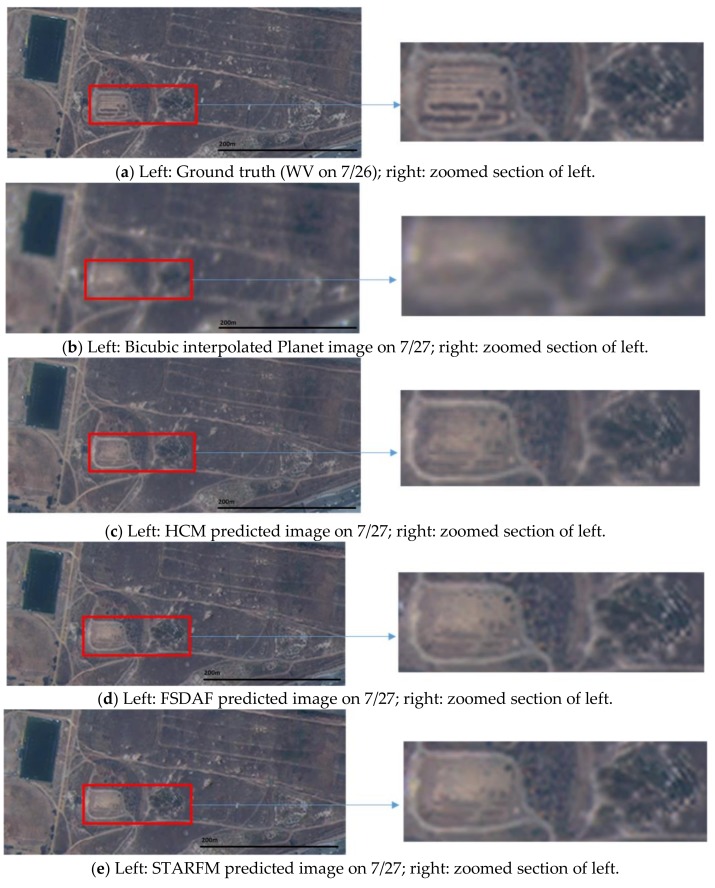
Comparison of different fused images with the ground truth (WV) and the low resolution Planet image at the prediction time. Field area.

**Figure 10:**
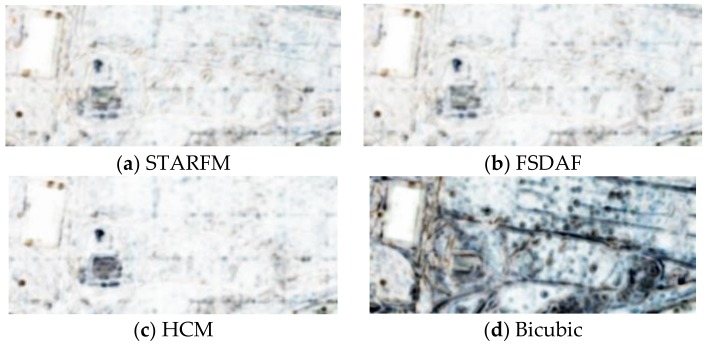
SSIM maps of the Field scenario using different methods. (**a**–**d**) are the maps for STARFM, FSDAF, HCM, and bicubic, respectively.

In this scenario, we used two Planet images collected at 19 July and 27 July and one WV image at 18 July to jointly predict a high resolution image at 27 July. Because there is no high resolution WV image at 27 July, we used the WV image at 26 July as the ground truth for generating the performance metrics. The prediction scenario is illustrated in Figure 8. From Table 6, one can see that the three fusion algorithms have improved the PSNRs by about 3 dBs over the bicubic interpolated Planet image. The SSIM values have also been improved by close to 50%. Other metrics have all been improved over the bicubic outputs. By closely inspecting the various images in Figure 9, the fused images can recover more fine details, which were blurred and hidden in the original Planet image at the prediction time. Figure 10 shows the SSIM maps of different methods. We can see that the improvement over bicubic is quite significant.

### 3.3. Scenario 3: Runway Area

**Figure 11:**
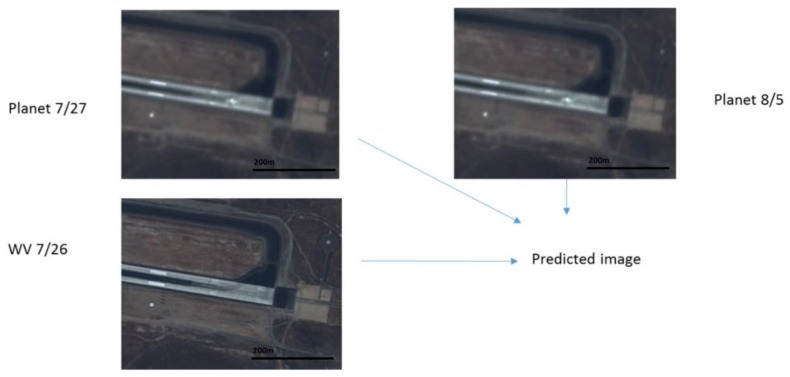
Two Planets images at 27 July and 5 August and one WV image at 26 July are fused to generate a prediction. The prediction is then compared to a WV image collected on 3 August 2017.

**Figure 12:**
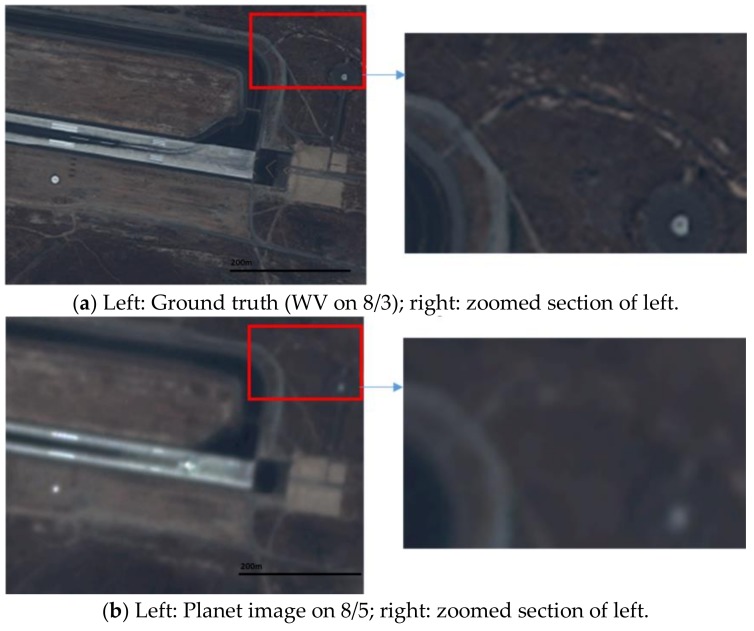

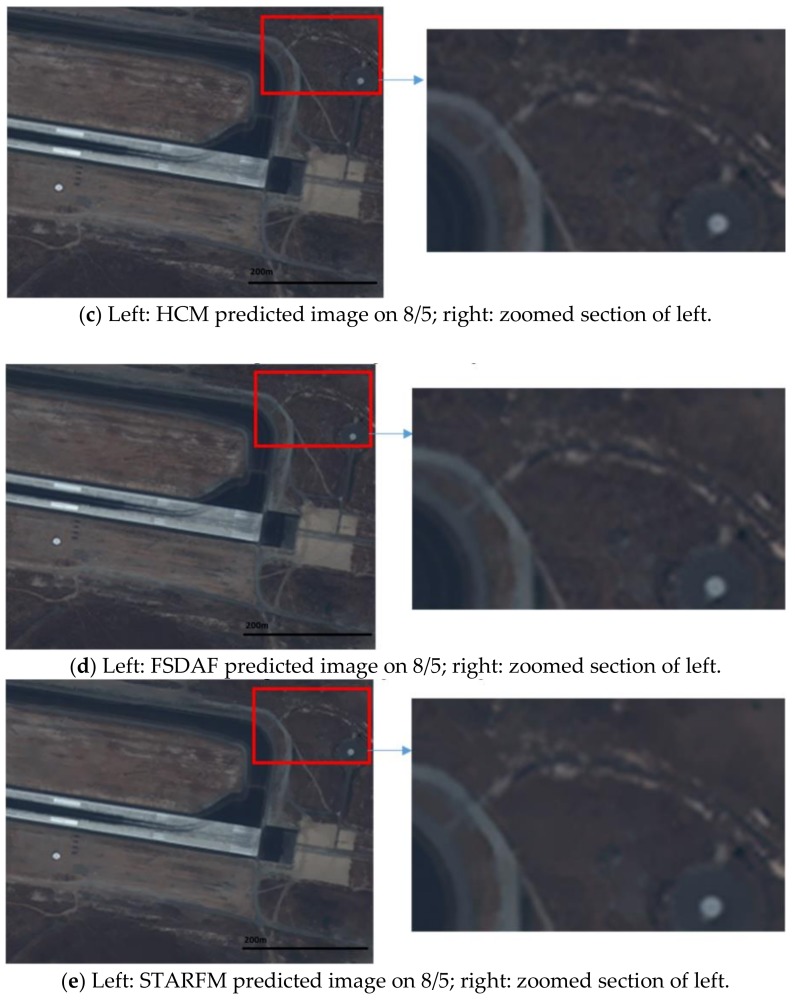
Comparison of different fused images with the ground truth (WV) and the low resolution Planet image at the prediction time. Runway area.

Here, we used two Planet images at 27 July and 5 August and one WV image at 26 July to generate a prediction at 5 August. Due to lack of a WV image at 5 August, we used the WV image at 3 August as the ground truth. Figure 11 illustrates the prediction scenario. The prediction performance metrics are summarized in Table 7. In terms of PSNR, all three fusion algorithms improved over the Planet image by 3 dBs. In terms of SSIM, the improvement is close to 10%. Other metrics show a similar trend as the above metrics. In terms of subjective evaluation, one can see from Figure 12 that the fusion algorithms can recover the fine details whereas the Planet image missed quite a few of the detailed features inside the red bounding box.

**Figure 13:**
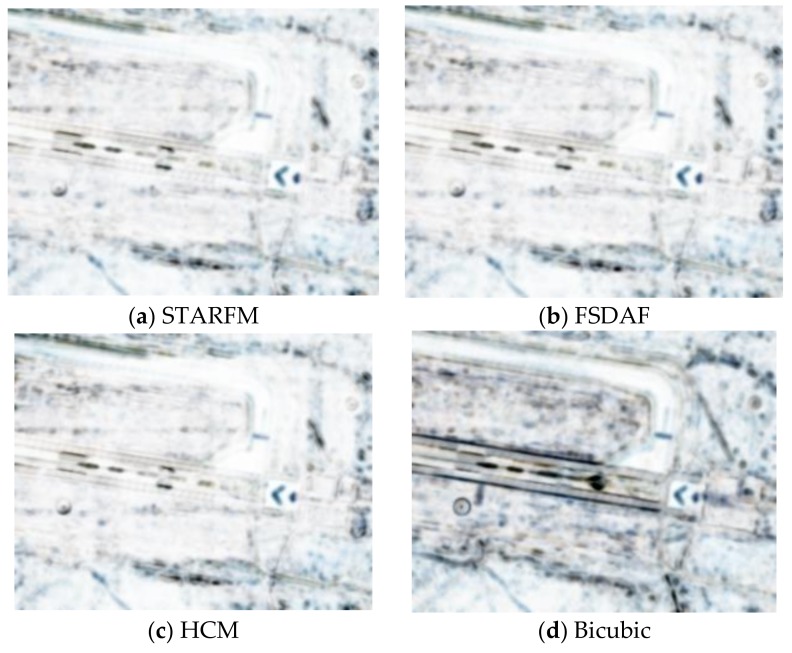
SSIM maps of the Runway scenario using different methods. (**a**–**d**) are the maps for STARFM, FSDAF, HCM, and bicubic, respectively.

Figure 13 shows the SSIM maps, which further corroborate that the HCM, FSDAF, and STARFM all have better performance than bicubic.

## 4. Discussion

The study in this paper is important because Planet images are collected using CubeSat’s and have more frequent revisit times, as compared to the WV images. However, the resolution of Planet is worse than that of WV images. The methods described in this paper can generate a high spatial resolution and high temporal resolution image series by fusing the two satellite images and will have many important applications, including border monitoring, damage assessment, etc. Decision makers can perform accurate situation assessment based on the fused data.

Although there are quite a few fusion studies on MODIS and Landsat images, no one has carried out a fusion study for Planet and WV images to the best of our knowledge. We presented three approaches to fusing the Planet and WV images. The approaches are representative algorithms in the literature. STARFM is well-known in the fusion of MODIS and Landsat. FSDAF incorporates clustering information to assist the prediction process, but requires more computations. HCM is a relatively simple and efficient algorithm for prediction. Based on the experimental results on three scenarios near a US-Mexico border area, the improvement of the three fusion approaches over the original Planet images is significant. In particular, the PSNR gains are close to 3 dB (Table 5, Table 6 and Table 7) for some of the three scenarios. Other performance metrics such as AD, RMSE, SAM, SSIM, Q2N, ERGAS, and CC all show significant improvement over the baseline (bicubic). In addition to the above metrics, we also generated SSIM index maps (Figure 7, Figure 10 and Figure 13), which also show significant visual improvement of the results from HCM, FSDAF, and STARFM over that of the bicubic method. We can also visually see that the prediction images from all of the algorithms can reveal some fine textures that are missing in the Planet images. Moreover, as can be seen from Figure 6, Figure 9 and Figure 12, the fused images have much better visual quality than the original Planet images.

It is also worth to mention that, from the results in Section 3, one can observe that none of the three methods can perform well under all scenarios. It is therefore important to have a library of algorithms for image fusion. The best algorithm should be selected for a given application.

Since the Planet images are top of atmosphere radiance (TOAR), we opted to work in radiance domain by also using WV-2 images (TOAR). The two types of images indeed have magnitude differences in the radiance domain. To overcome this, we carried out some preprocessing. In pansharpening papers, a common practice has been widely used, which is to apply histogram matching [16,17] between pan band and the multispectral bands. Here, we adopted a similar strategy in order to work directly in radiance domain. The idea is actually very simple. We used W1 as the reference and applied histogram matching to P1 and P2 so that P1 and P2 are matched to W1. Since W1 and W2 are collected at the same time of the day with the same altitude (the Worldview satellite flies over the same area at roughly the same time of the day), they have roughly the same radiance. We then learn the mapping between the adjusted P1 and P2. Finally, we perform the mapping from W1 to W2 by using the learned mapping earlier.

It should be noted that, even if we work directly in the reflectance domain, there is no guarantee that the reflectance values between the Planet and Worldview images will be the same. We may still need to perform some histogram matching after atmospheric compensation. This is because the atmospheric compensation procedures in generating the Worldview reflectance images are proprietary. Digital Globe has a proprietary software known as GBDX (https://gbdxdocs.digitalglobe.com/docs/advanced-image-preprocessor), which contains atmospheric compensation algorithms. If we use FLAASH in ENVI to generate reflectance images for Planet, the two sets of reflectance images resulting from different atmospheric compensation algorithms may still be slightly different and hence histogram matching may still be required after atmospheric compensation. 

Because Worldview images are not collected at nadir for most of the images, the registration and alignment with Planet images should be done carefully. In this research, we performed the alignment manually. One future direction is to develop an automated alignment program that will significantly reduce manual labor.

The image compatibility issue between different satellite images together with the image alignment issue require more in depth studies in the future. The image alignment is non-trivial. Here, our goal is on image fusion algorithm assessment where we assume that the registration is done and the images (regardless whether they are radiance or reflectance) have similar histograms.

Another direction is to apply the high temporal high spatial resolution images to some applications such as damage assessment due to flooding, fire, hurricane, tsunami, etc.

## 5. Conclusions

In this paper, we applied three spatiotemporal data fusion approaches for forward predicting images with high spatial and high temporal resolutions from Planet and WV images. The performance of the three approaches is comparable using actual WV and Planet images. Compared to other fusion approaches such as STAARCH and ESTARFM, the methods tested in this study do not require two pairs of Planet and WV images and are more appropriate for forward prediction. 

## Figures and Tables

**Table 1:** Means and stds of Red band of two image pairs for the three scenarios in Section 3.

		Image	CANAL	FIELD	RUNWAY
Red Band	MEAN	PSE	0.302202	0.383428	0.269307
		WVE	0.303498	0.381238	0.263837
					
		PSL	0.244982	0.356089	0.268762
		WVL	0.244404	0.354388	0.243716
					
	STD	PSE	0.096934	0.084006	0.093859
		WVE	0.099734	0.085258	0.093664
					
		PSL	0.082842	0.089564	0.093986
		WVL	0.083615	0.089764	0.088597

**Table 2:** Means and stds of Green band of two image pairs for the three scenarios in Section 3.

		Image	CANAL	FIELD	RUNWAY
Green Band	MEAN	PSE	0.303195	0.390444	0.273252
		WVE	0.304829	0.387911	0.26864
					
		PSL	0.251461	0.354358	0.272753
		WVL	0.250843	0.353061	0.250539
					
	STD	PSE	0.090393	0.064076	0.093683
		WVE	0.093879	0.064833	0.09259
					
		PSL	0.075665	0.070846	0.093826
		WVL	0.07582	0.069963	0.085556

**Table 3:** Means and stds of Blue band of two image pairs for the three scenarios in Section 3.

		Image	CANAL	FIELD	RUNWAY
Blue Band	MEAN	PSE	0.321027	0.431879	0.295805
		WVE	0.322543	0.42958	0.293019
					
		PSL	0.269751	0.392876	0.295402
		WVL	0.268997	0.390579	0.275519
					
	STD	PSE	0.084322	0.049597	0.0866
		WVE	0.086584	0.050008	0.084989
					
		PSL	0.069209	0.055778	0.086771
		WVL	0.06806	0.055536	0.077771

**Table 4:** Band specifications of Planet and Worldview images.

Planet Image (3.125 m Resolution) with 4 Bands		Worldview-2 Image (2 m Resolution) with 8 Bands			
Blue	455–515 nm	Coastal	400–450 nm	Red	630–690 nm
Green	500–590 nm	Blue	450–510 nm	Red Edge	705–745 nm
Red	590–670 nm	Green	510–580 nm	Near-IR1	770–895 nm
NIR	780–860 nm	Yellow	585–625 nm	Near-IR2	860–1040 nm

**Table 5:** Comparison of prediction results of using Planet and WV images for the scenario depicted in Figure 5. Bold characters indicate the best performing method for that row.

**RGB**					**R**				
**Canal**					**Canal**				
	STARFM	FSDAF	HCM	Bicubic		STARFM	FSDAF	HCM	Bicubic
RMSE	0.0377	0.0373	**0.0373**	0.0403	RMSE	0.0402	0.0403	**0.0388**	0.0443
PSNR	28.4721	28.5595	**28.5760**	27.9017	PSNR	27.9065	27.8942	**28.2132**	27.0623
AD	0.0221	0.0222	**0.0221**	0.0259	AD	**0.0241**	0.0245	0.0242	0.0303
CC	0.4384	**0.4411**	0.4368	0.4229	CC	0.8499	0.8487	**0.8599**	0.8199
SAM	2.1033	**2.0274**	2.3677	2.3576	SSIM	**0.8230**	0.8200	0.8138	0.6687
SSIM	0.8491	**0.8493**	0.8350	0.7366	ERGAS	16.7615	**16.9272**	16.2325	18.5180
ERGAS	15.2969	15.2785	**15.0919**	16.4045	Q2N	0.7844	**0.7806**	0.7906	0.6755
Q2N	0.7795	**0.7814**	0.7797	0.6737					
(**a**) Metrics for all 3 bands					(**b**) Metrics for Red band				
**G**					**B**				
**Canal**					**Canal**				
	STARFM	FSDAF	HCM	Bicubic		STARFM	FSDAF	HCM	Bicubic
RMSE	0.0374	0.0372	**0.0363**	0.0396	RMSE	0.0353	**0.0342**	0.0366	0.0364
PSNR	28.5357	28.5890	**28.8083**	28.0401	PSNR	29.0499	**29.3102**	28.7308	28.7733
AD	0.0220	0.0222	**0.0208**	0.0255	AD	0.0204	**0.0201**	0.0214	0.0220
CC	0.8089	0.8104	**0.8206**	0.7836	CC	0.7692	**0.7766**	0.7567	0.7403
SSIM	**0.8443**	0.8429	0.8380	0.7148	SSIM	**0.8448**	0.8443	0.8178	0.7413
ERGAS	15.3994	15.4212	**14.9527**	16.3709	ERGAS	13.5607	**13.2648**	14.0079	14.0142
Q2N	0.7592	0.7546	**0.7811**	0.6686	Q2N	0.7237	0.7177	**0.7284**	0.6468
(**c**) Metrics for Green band					(**d**) Metrics for Blue band				

**Table 6:** Comparison of prediction results of using Planet and WV images for the scenario depicted in Figure 8.

**RGB**					**R**				
**Field**					**Field**				
	STARFM	FSDAF	HCM	Bicubic		STARFM	FSDAF	HCM	Bicubic
RMSE	0.0302	0.0286	**0.0266**	0.0479	RMSE	0.0337	0.0321	**0.0308**	0.0560
PSNR	30.4069	30.8705	**31.5069**	26.3870	PSNR	29.4494	29.8801	**30.2383**	25.0355
AD	0.0206	0.0194	**0.0168**	0.0335	AD	0.0233	0.0220	**0.0196**	0.0400
CC	0.4967	0.5025	**0.5121**	0.4223	CC	0.9300	0.9360	**0.9394**	0.8047
SAM	1.4415	1.2646	**1.0583**	1.7460	SSIM	0.8661	0.8723	**0.8926**	0.5412
SSIM	0.8902	0.8966	**0.9156**	0.6574	ERGAS	9.5117	9.0515	**8.7089**	15.8107
ERGAS	8.3115	7.8876	**7.3729**	13.2696	Q2N	0.8602	0.8712	**0.8967**	0.5391
Q2N	0.8439	0.8559	**0.8929**	0.5461					
(**a**) Metrics for all 3 bands					(**b**) Metrics for Red band				
**G**					**B**				
**Field**					**Field**				
	STARFM	FSDAF	HCM	Bicubic		STARFM	FSDAF	HCM	Bicubic
RMSE	0.0286	0.0274	**0.0259**	0.0464	RMSE	0.0279	0.0260	**0.0225**	0.0400
PSNR	30.8846	31.2412	**31.7407**	26.6616	PSNR	31.0748	31.7023	**32.9749**	27.9594
AD	0.0195	0.0187	**0.0166**	0.0328	AD	0.0189	0.0176	**0.0141**	0.0276
CC	0.9180	0.9240	**0.9292**	0.7820	CC	0.8760	0.8895	**0.9150**	0.7418
SSIM	0.8827	0.8868	**0.9047**	0.6042	SSIM	0.8619	0.8713	**0.9065**	0.6407
ERGAS	8.0951	7.7694	**7.3551**	13.1633	ERGAS	7.1583	6.6595	**5.7564**	10.2467
Q2N	0.8522	0.8607	**0.8885**	0.5361	Q2N	0.8054	0.8209	**0.8782**	0.5155
(**c**) Metrics for Green band					(**d**) Metrics for Blue band				

**Table 7:** Comparison of prediction results of using Planet and WV images for the scenario depicted in Figure 11.

**RGB**					**R**				
**Runway**					**Runway**				
	STARFM	FSDAF	HCM	Bicubic		STARFM	FSDAF	HCM	Bicubic
RMSE	0.0327	**0.0316**	0.0329	0.0457	RMSE	0.0344	**0.0333**	0.0348	0.0489
PSNR	29.7128	**30.0081**	29.6460	26.8029	PSNR	29.2637	**29.5449**	29.1655	26.2078
AD	0.0231	**0.0218**	0.0231	0.0304	AD	0.0255	**0.0242**	0.0256	0.0336
CC	0.5300	**0.5301**	0.5293	0.5031	CC	0.9534	**0.9534**	0.9517	0.8967
SAM	1.3649	1.3469	**1.3208**	1.8684	SSIM	**0.8451**	0.8451	0.8417	0.7209
SSIM	**0.8896**	0.8892	0.8862	0.7903	ERGAS	14.0951	13.6461	**13.2019**	20.0384
ERGAS	12.8138	12.3881	**12.0533**	17.9663	Q2N	0.6589	0.6783	**0.7852**	0.5326
Q2N	0.7932	0.8020	**0.8081**	0.6833					
(**a**) Metrics for all 3 bands					(**b**) Metrics for Red band				
**G**					**B**				
**Runway**					**Runway**				
	STARFM	FSDAF	HCM	Bicubic		STARFM	FSDAF	HCM	Bicubic
RMSE	0.0323	**0.0313**	0.0325	0.0463	RMSE	0.0312	**0.0301**	0.0315	0.0415
PSNR	29.8063	**30.0904**	29.7745	26.6800	PSNR	30.1111	**30.4357**	30.0453	27.6414
AD	0.0227	**0.0214**	0.0226	0.0304	AD	0.0211	**0.0197**	0.0211	0.0271
CC	0.9566	**0.9568**	0.9557	0.9023	CC	0.9528	**0.9531**	0.9513	0.9088
SSIM	**0.8758**	0.8757	0.8737	0.7656	SSIM	**0.8908**	0.8894	0.8856	0.8023
ERGAS	12.8810	12.4665	**12.0869**	18.4612	ERGAS	11.3131	10.8981	**10.7453**	15.0338
Q2N	0.6759	0.7228	**0.8018**	0.5914	Q2N	0.6578	0.6973	**0.7830**	0.5854
(**c**) Metrics for Green band					(**d**) Metrics for Blue band				
